# Highly conductive and pure gold nanostructures grown by electron beam induced deposition

**DOI:** 10.1038/srep34003

**Published:** 2016-09-26

**Authors:** Mostafa M. Shawrav, Philipp Taus, Heinz D. Wanzenboeck, M. Schinnerl, M. Stöger-Pollach, S. Schwarz, A. Steiger-Thirsfeld, Emmerich Bertagnolli

**Affiliations:** 1 Institute of Solid State Electronics, Vienna University of Technology, Vienna 1040, Austria; 2 University Service Center for Transmission Electron Microscope (USTEM), Vienna University of Technology, Vienna 1040, Austria

## Abstract

This work introduces an additive direct-write nanofabrication technique for producing extremely conductive gold nanostructures from a commercial metalorganic precursor. Gold content of 91 atomic % (at. %) was achieved by using water as an oxidative enhancer during direct-write deposition. A model was developed based on the deposition rate and the chemical composition, and it explains the surface processes that lead to the increases in gold purity and deposition yield. Co-injection of an oxidative enhancer enabled Focused Electron Beam Induced Deposition (FEBID)—a maskless, resistless deposition method for three dimensional (3D) nanostructures—to directly yield pure gold in a single process step, without post-deposition purification. Gold nanowires displayed resistivity down to 8.8 μΩ cm. This is the highest conductivity achieved so far from FEBID and it opens the possibility of applications in nanoelectronics, such as direct-write contacts to nanomaterials. The increased gold deposition yield and the ultralow carbon level will facilitate future applications such as the fabrication of 3D nanostructures in nanoplasmonics and biomolecule immobilization.

Gold nanostructures are a promising candidate for applications in plasmonics, biosensors, and electrical contacts owing to their excellent dielectric function, biocompatibility, and electrical properties^1^,^2^,^3^. However, most applications require either highly pure or highly conductive gold. To fabricate such nanostructures, there are several nanofabrication techniques, such as conventional photolithography and electron beam lithography, that are used in conjunction with metal layer deposition. Nevertheless, these methods fabricate nanostructures only on planar, smooth surfaces and require a complex multi-step process sequence to pattern material. They are further limited by the obligatory use of photoresist. To overcome these fabrication limitations, an additive direct-write lithography that can generate nanostructures without the need for resist or a photomask would be beneficial. Focused Ion Beam-Induced Deposition (FIBID) has become popular as direct-write nanolithography^4^. However, Ga^+^ implantation during deposition and atomic mixing are major downsides of the FIBID process^5^.

Focused Electron Beam-Induced Deposition (FEBID) is another such direct-write nanofabrication technique, in which an electron beam of a scanning electron microscope is used to locally dissociate precursor molecules, and thereby deposit the desired material on the nanometre scale^6^,^7^. This technique has already shown its potential for different applications. For example, various materials, including iron^8^,^9^,^10^, cobalt^11^,^12^, tungsten^13^, and platinum^14^,^15^ have already been deposited for applications such as nanomagnet logic^16^, magnetic force microscopy^17^,^18^,^19^, hall sensors^11^,^20^, patterning stencil masks^21^, separating nanoparticles^22^,^23^, and plasmonics^24^,^25^. FEBID has also been used to deposit gold nanostructures for metal-oxide semiconductor capacitors^26^ and nanoantennas for surface-enhanced Raman spectroscopy^27^,^28^.

However, FEBID gold nanostructures are generally fabricated using organometallic precursors such as AuMe_2_(acac) or AuMe_2_(tfac), which contain a large amount of carbon. Therefore, these structures suffer from a carbon contamination problem. Generally, commercially available precursor produces gold nanostructures with a metal purity around 25 at. %^27^,^29^. Such low metal content is the real challenge for a wider application of FEBID gold structures. Previously, carbon-free precursors have been used, which show high metal content^30^,^31^. However, these precursors became unpopular owing to their thermal instability, resulting in premature decomposition. Different post-deposition purification techniques^32^,^33^,^34^, such as annealing^35^,^36^, substrate heating^37^,^38^, laser-assisted FEBID^39^, electron beam curing^40^ and post-deposition exposure to water^41^,^42^ and oxygen flux^43^ have been utilized to purify various FEBID structures. However, there is no *in situ* deposition procedure that can deposit highly pure and conductive FEBID Au in a single process step.

This work presents a specially enhanced process to deposit highly pure and conductive FEBID Au structures directly, without any post-deposition purification approach. This *in situ* purification is achieved by the injection of water vapor as a second gas, together with the organometallic precursor simultaneously, during the deposition of Au nanostructures. This injection leads to the highest gold purity obtained so far from a direct deposition. This atomic composition was verified by i) Scanning Electron Microscopy (SEM) Energy Dispersive Spectroscopy (EDX), ii) Transmission Electron Microscopy (TEM) EDX and iii) TEM Electron Energy Loss Spectroscopy (EELS). The resistivity of the deposit was measured using the standard I-V characteristics. Finally, a model is discussed, showing the actual deposition of gold and carbon.

## Results

**Figure 1:**
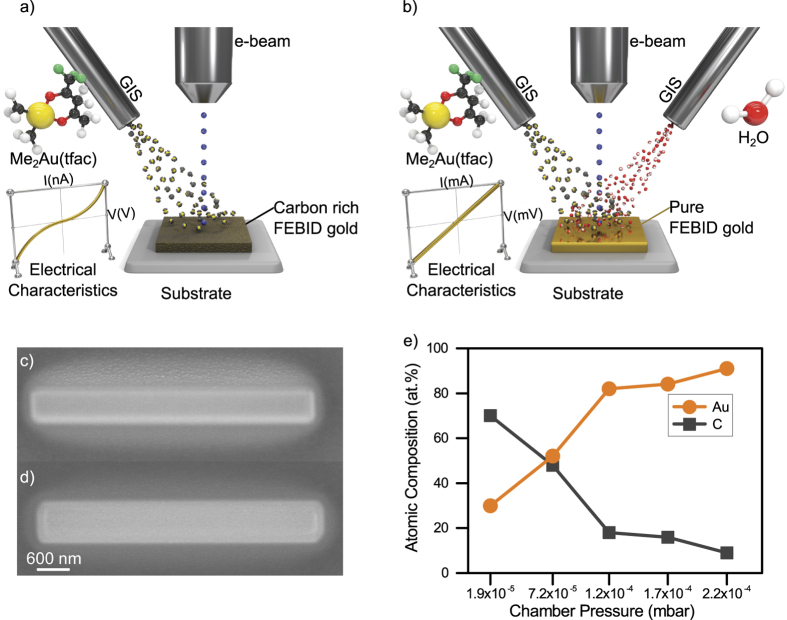
Specially enhanced FEBID process. (**a**) Conventional FEBID Au process (**b**) Water-assisted FEBID Au process (**c**) Conventional FEBID Au structure, (**d**) Water-assisted FEBID Au, (**e**) Atomic composition obtained from SEM EDX for different chamber pressures.

In a typical FEBID process shown in Fig. 1a, there are two major factors that result in low metal content of the deposit: i) carbon deposition from the residual gas in the SEM chamber and ii) carbon originating from the ligands of metalorganic precursors. To reduce the contribution of carbon deposition from residual gas inside the SEM, the chamber was cleaned prior to deposition utilizing highly energetic ultraviolet light combined with highly reactive ozone^44^.

The precursor molecule dimethylgold (III) trifluoroacetylacetonate has a carbon-to-gold ratio of 7:1. In a typical deposition, this precursor generally delivers ~15–30 at. % gold content^40^,^45^. To deposit a highly pure gold nanostructure from this precursor, a second gas was injected simultaneously during the deposition as presented in Fig. 1b. This second gas works as an oxidative enhancer. Such an oxidative enhancement may reduce or prevent carbon contamination during the deposition. Owing to its high polarity and high adsorption coefficient on most surfaces, water was chosen as the oxidative species. This study focused on whether and to what extent this additional oxidative enhancement can improve the chemical purity of the gold deposit.

### Scanning Electron Microscopy (SEM) Investigations

A SEM image of the conventional FEBID Au and water-assisted FEBID Au are presented in Fig. 1c,d. To investigate the effect of water on the deposition process, several depositions were performed with increasing water content. Starting from a base pressure of ~6 × 10^−7^ mbar, the injection of the gold precursor has led to an increase in the chamber pressure by ~1.84 × 10^−5^ mbar (i.e., total chamber pressure of ~1.90 × 10^−5^ mbar). In the case of water injection, the chamber pressure was again increased by 2.01 × 10^−4^ mbar (i.e., total chamber pressure of 2.20 × 10^−4^ mbar), while keeping the gold pressure constant. Because the nozzle geometry was comparable, in the ultimate case, a 10-fold flow of water is injected relative to the metalorganic gold precursor. The image in Fig. 1d indicates that the water addition may also influence the geometry of the deposit as well as the geometry of the halo. If this is a real chemical effect or just a visual artifact due to different contrast settings or different secondary electron-yield depending on the material purity and sample drift during deposition- is still under investigation.

For the deposited areas, the atomic composition obtained via SEM EDX depending on the chamber pressure is presented in Fig. 1e. The first data point reflects the situation in which no additional water was injected and shows a gold content of only 30 at. %. Injecting the water (and maintaining) up to a total chamber pressure of ~7.2 × 10^−5^ mbar and resulted in a gold content of 52 at. %. A further increase in water partial pressure results in a chamber pressure of ~1.2 × 10^−4^ mbar and leads to a gold content of 82 at. %. Maximum gold purity of 91 at. % was obtained at a chamber pressure of ~2.2 × 10^−4^ mbar. Figure 1e indicates that the atomic composition of the FEBID Au structure can be tuned by varying the water pressure during deposition. An EDX map and a line scan obtained from a typical square deposition are shown in Supplement 1a,b, which proves that the entire area of the deposited material has homogeneously high gold content.

In the next step, the atomic composition of FEBID Au that was specifically enhanced by water injection was compared with a conventional sputtered gold deposition. Generally, sputter-deposited films have a composition that is close to the source material (99.99 at. %). That is why this conventional method is used in the industry to deposit pure films of desired material. Before obtaining the EDX from sputtered gold, the sample was cleaned with oxygen plasma. The sputtered gold resulted in purity from 76 at. % to 89 at. % with an average value of 83 at. % from 19 different measurement regions.

Quantification of the accurate gold content by EDX is considered to be a critical issue owing to the error-prone algorithm of background correction. Therefore, the EDX system was calibrated with a 99.99 at. % pure sputtered layer showing that an EDX read-out value of 83 at. % actually corresponds to 99.99 at. % pure gold. This result is in perfect agreement with the work and prediction of Mulders *et al*.^46^,^47^,^48^. This calibration and the literature suggest that oxidative assisted FEBID process produced pure gold nanostructures that are comparable to other methods.

This result also indicates that one step deposition and “*in-situ*” purification of FEBID gold work simultaneously and produce highly pure gold structures.

### Transmission Electron Microscopy (TEM) Investigations

**Figure 2:**
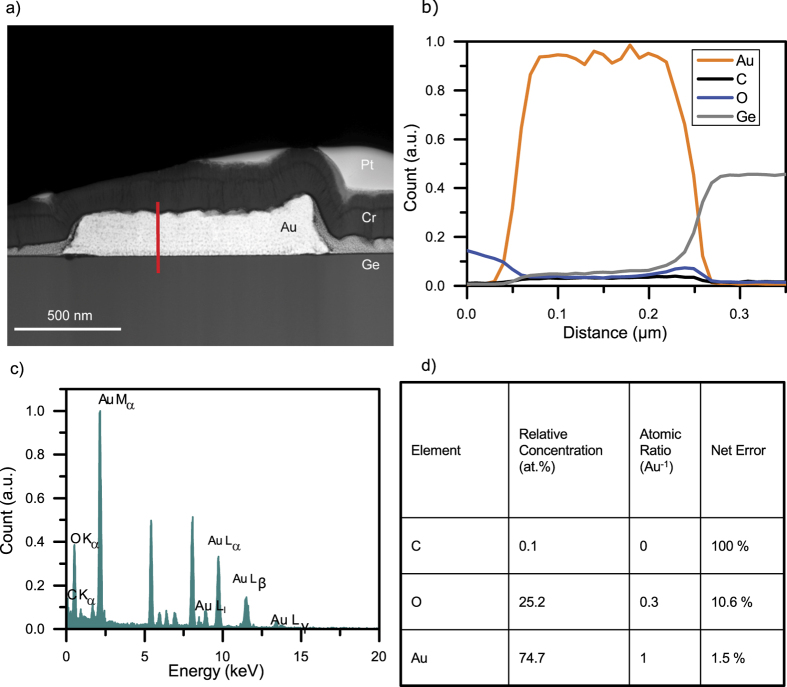
TEM EDX investigations. (**a**) HAADF STEM image of the assisted FEBID gold structure. The image reveals a mass contrast among the germanium substrate, the FEBID gold deposit, the sputtered chrome layer, and the applied FEBID platinum layer. The grey area between the chrome layer and Ge is halo deposition from secondary electrons. (**b**) TEM EDX spectrum of water-assisted FEBID gold structure. (**c**) TEM EDX line scan along the z-axis of the deposition (marked with a red line in **a**). (**d**) TEM EDX quantification results.

To confirm the SEM findings, extensive TEM analysis was conducted on the water-assisted FEBID Au nanostructures. As test structures, square shape FEBID-Au nanostructures were deposited on a Ge substrate, using the parameter set mentioned in the method section. The TEM lamella of this FEBID-Au nanostructure was prepared by FIB milling. Prior to FIB milling, a capping layer of chromium was sputtered on the deposit with a nominal thickness of 150 nm. This layer shielded the gold deposit from ion and (even more importantly) electron irradiation during the focused ion beam processing of the cross section. Hence, electron beam-induced curing is effectively excluded, and the Monte Carlo simulation shown in Supplement 2a,b confirms that 5 keV primary electron beam energy cannot penetrate the 150 nm thick protective chrome layer. In addition, prior to FIB processing, a protective platinum layer was also deposited using FEBID. In the bright field TEM image presented in Supplement 2c, all four different layers of Ge, FEBID gold, sputtered chrome layer, and a FEBID Pt layer can be clearly distinguished The selected area diffraction (SAED) shown in Supplement 2d was obtained from an area approximately 100 nm around the centre of the Au-FEBID deposit. The SAED shows that the first diffraction ring is located 4.19 nm^−1^ from the un-diffracted spot in the reciprocal space; this corresponds to a distance of 0.238 nm for the 111 gold plane. This defined diffraction ring confirms that the deposited gold has polycrystalline behaviour. A High-Angle Annular Dark-Field (HAADF) scanning transmission electron microscopy (STEM) image obtained of the FEBID-Au is shown in Fig. 2a. The top layer corresponds to homogeneous FEBID platinum, appearing dimmer relative to the deposited gold structure. The gold deposit shows small dark spots due to the coalescing carbon clusters entrapped in the gold. The halo deposition around the primary deposit shows the typical EBID gold crystallites embedded in a carbonaceous matrix^40^,^49^,^50^. The sputtered chrome layer appears to be darker than the heavier gold and platinum deposits. The grey area between the protective chrome layer and Ge substrate is a halo deposition from secondary electrons. The location of the grey layer, its decreasing thickness from the scan area to the outside are the typical characteristics of a halo deposition in FEBID process. This halo can be removed by post-deposition Ar^+^ milling^9^.

A TEM EDX spectrum and a line scan along the z-axis (shown in red line) of the deposition are presented in Fig. 2b,c. The TEM EDX measurement of the FEBID-Au indicates a gold content of 74.2 at. %, whereas carbon was present in the structure in insignificant amounts. The oxygen content (detailed in Supplement 3) was relatively high when compared to the measurements shown in Supplement 1b. The EDX spectrum shows some unexpected peaks that are attributed to copper, iron, cobalt, and chrome, and those are not related to the FEBID Au process. These signals are generated mainly from applied chrome coating or from the microscope itself. Whereas these signals were considered during the background correction, they were corrected in the final quantification of gold, carbon, and oxygen ratio (the final interpretation focused only on the quantification of gold, carbon, and oxygen). Moreover, the essential assigned peaks, arranged by energies, are listed in Supplement 3a. The EDX line scan was performed along the height axis, starting from the chrome protection layer down to the germanium substrate. As expected, the sputtered chrome layer is oxygen rich because chrome oxidizes easily. The oxygen count is increased, both at FEBID deposition and pure Ge substrate. This can explain the relatively high oxygen content detected in the EDX measurement. A further increase in the oxygen count is evident at the interface between the gold and germanium. This is due to the formation of germanium oxide in the presence of water during the initial phases of the FEBID process. The low carbon count mostly remains constant along the height (z) axis, and the gold count is very significant and much higher in comparison to all other counts.

**Figure 3:**
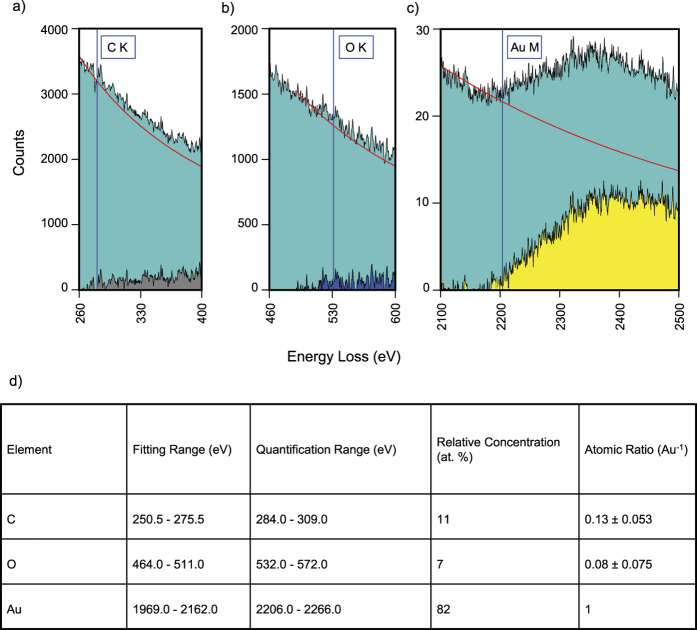
Detailed parts of the TEM EELS spectrum. The acquired raw count is shown in green, the fitted exponential background is drawn in red, and the resulting background corrected signal is shown. The shown energy range in (**c**) is broader compared with (**a,b**) because of the delayed edge characteristic of the gold M signal. (**d**) EELS quantification results and parameters.

TEM EELS analysis was performed to further investigate the chemical composition, especially of light elements. The full spectrum obtained from an energy range 0 to 2800 eV is shown in Supplement 3b. The carbon K edge is at 284 eV energy loss, whereas the oxygen K edge is at 532 eV. The gold M edge is marked at 2206 eV. A detailed section of the full spectra for carbon, oxygen, and gold are shown in Fig. 3a–c. The fitted background is shown in red, whereas the subtracted signal is shown in grey for carbon, blue for oxygen, and yellow for gold. The quantification shown in Fig. 3d demonstrates, that the deposit has a gold content of 82 at. %. Summarizing, TEM investigation indicates that water-assisted FEBID Au has a very high gold concentration.

The atomic compositions obtained from SEM EDX (which is more reliable at high mass numbers) and TEM EELS (which is more reliable at low mass numbers) are presented in Table 1. Both analysis methods equally indicate a high gold content of the FEBID Au structures that were deposited using water as an additional gas. This gold content is above 80 at. %, and it is indeed the highest gold content obtained so far from a one-step direct FEBID process while using a commercial metal organic gold precursor. On a more general scale, comparing with all metals, the highest metal content of 95 at. % was achieved earlier from a cobalt metal organic precursor^51^.

### Electrical Resistivity Measurements

For various nanoelectronic applications, a highly pure and highly conductive gold structure is essential. High conductivity is also a measure of the metal’s purity. This is why, in the final step, electrical resistivity measurements of highly pure FEBID Au were performed. At the beginning, 4 point-probe contacts made of Au were fabricated using conventional photolithography and a lift-off process. A FEBID-Au nanowire was then deposited across the 4 metal contacts, which were 2 μm wide and 150 nm high and had a spacing of 2 μm. Two different sets of depositions were performed on separate samples. First, a conventional FEBID Au nanowire was deposited without an oxidative enhancer (~1.4 × 10^−5^ mbar). Second, FEBID Au was deposited using water as an oxidative enhancer (~2.2 × 10^−4^ mbar). The height of the deposited water-assisted Au nanowires was 30 nm, determined from an AFM height profile. For electrical characterization, the corresponding I-V characteristics of both FEBID-Au nanowires were measured. During the I-V measurement, voltage was applied to the outer contacts, inducing a current across the device being tested. The current values of the two inner measurement electrodes were set to zero, and the voltage across them was measured current-free.

**Figure 4:**
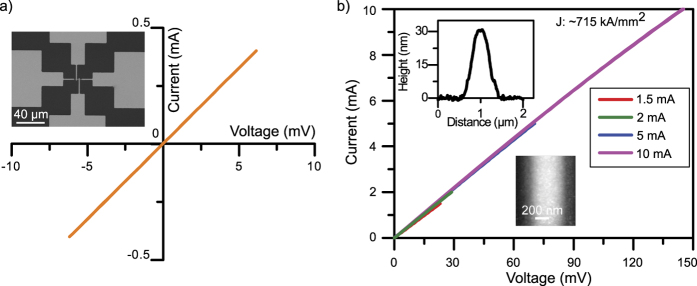
Electrical resistivity measurements. (**a**) I-V characteristics of the “*in-situ*” purified FEBID Au nanowire. The inset in (**a**) shows an SEM image of the used four-point contacts. (**b**) A measurement series with increasing maximum current from 1.5 mA to 10 mA is shown. The insets in (**b**) show an AFM image and height profile of the deposited structure.

For soft testing, the current range through the nanowire was gradually increased. First, low current values limited to 8.2 μA were chosen to avoid current-induced effects, such as electromigration and thermal annealing. The voltage drop between the inner measurement electrodes increased with the increasing current. This resulted in a lower noise level of the calculated resistance, where increasing current ranges were applied until failure (20 mA) of the FEBID-Au nanowire occurred. Conventional FEBID Au (without an oxidative enhancer) resulted in a tunnelling type conductance, as presented in Supplement 4. This also reveals a very high resistivity of 1 Ω cm, which is an indicator of a poor conductor. The conductivity measurements of water-assisted FEBID Au are presented in Fig. 4a,b. Both I-V graphs correspond to ideal ohmic behaviour. More than ten measurements resulted in resistivities from 10.9 μΩ cm down to a best case of 8.8 μΩ cm. To our knowledge, the highest FEBID gold conductivity was achieved by post-deposition purification, resulting in a resistivity of 17 μΩ cm^43^. For comparison, the bulk resistivity of gold is 2.2 μΩ cm^43^. With an oxidative enhancement during Au deposition, the resistivity of gold was just 4-fold higher than that of pure gold. This is, by far, the highest-conductivity measurement of FEBID Au obtained so far. The current density of the wire is ~715 kA/mm^2^, which is high enough for any typical nanoelectronic device application.

## Discussion

As shown by the SEM and TEM investigations, it is possible to deposit highly pure FEBID gold directly, without any post-deposition purification. To understand the mechanism underlying such deposition, a model was derived from the deposited volume and composition. This model proposes an explanation for the purification effects, together with the deposition rates.

To develop the model, the height of the deposited Au structures, deposited with different water pressure, was obtained from Atomic Force Microscopy (AFM). For the same deposits, the chemical composition was determined by SEM EDX. Owing to the different densities, the main components Au, C, and O contribute differently to the measured height than at. % measured by EDX. It should be noted that a gold atom takes more space than a carbon atom. Hence, the vol. % values were calculated based on the best obtained chemical composition and using following densities: Au 19.30 g/cm^3^ and graphitic C 2.26 g/cm^3^. For O, the molar mass/density was 6.23 g/mol which is an approximation that is mathematically derived from oxygen in solid compound. The total height of a deposit can be regarded as the sum of the volume contributions of the deposited Au atoms, C atoms and O atoms. This difference between the elemental composition (at. %) and the volume contributions (vol. %) is illustrated by Supplement 5, which shows the height of the structure and the corresponding Au, C, and O contributions in at. % (left bar) and the same height with the Au, C, and O contribution to this height as vol. % (right bar) for each chamber pressure. A detailed calculation of the specific volume can be found in Supplement 6.

**Figure 5:**
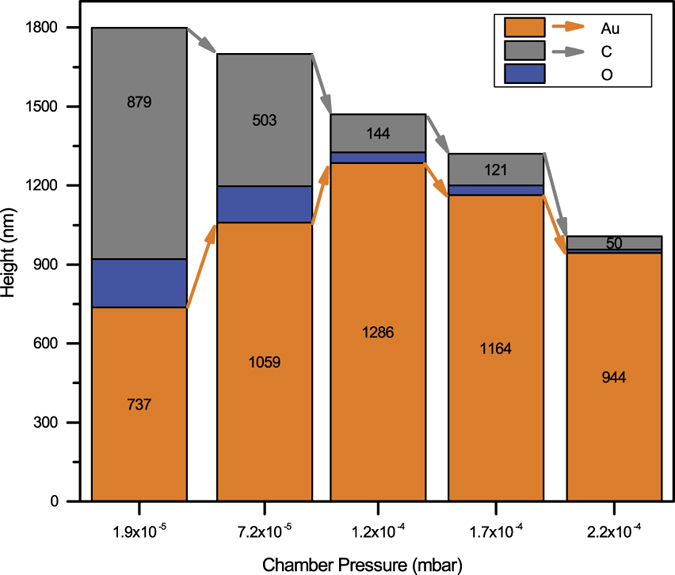
Height and volume contribution of the deposited gold (amber), oxygen (blue), and carbon (grey). An explanation of the calculation is shown in Supplement 6.

Figure 5 illustrates the height of the deposited gold structure, as obtained at different levels of oxidative enhancement. Except for the varying water injection, all other deposition parameters were maintained to be identical. Every height bar in Fig. 5 also reflects the volume contribution of the deposited gold (amber), oxygen (blue), and carbon (grey), based on the previously calculated vol. %. In short, the height of the bar represents the deposition rate, and the distribution of the areas for gold (amber), oxygen (blue), and carbon (grey) reflects their volume contribution.

Figure 5 shows that the total height of the deposited material constantly decreases with increasing water addition. The most remarkable fact is – the contribution of the gold to the total height (which is also the contribution of gold to the deposited volume) constantly increases for the first 3 depositions up to a total pressure of 1.2 × 10^−4^ mbar. In other words, when the deposition rate for the composite decreases, the deposition rate of the gold itself increases.

Conventional FEBID Au without water injection (chamber pressure: ~1.9 × 10^−5^ mbar) consists of 62 at. % carbon, which corresponds to 49 vol. % and is equivalent to ~879 nm of the height of the deposited volume. A gold content of 25 at. % corresponds to 40.9 vol. % Au, and this is equivalent to ~737 nm of the height of the deposited volume. The oxygen in the deposit could theoretically be trapped in or bonded to either the gold or the carbon. Because depositions with higher gold content also resulted in a much lower carbon deposition (chamber pressure: ~2.2 × 10^−4^ mbar), it is speculated that the oxygen is mainly bound to the carbon as a hydroxyl group or ketone group. The latter would be reasonable because one precursor molecule contains two ketone groups in every trifluoroacetylacetonate ligand. Based on the space consumed by oxygen in a solid compound, the volume of the bound oxygen contributes to ~183 nm of the total height of the deposit.

A post-deposition cleaning thought experiment can be imagined on this 1800 nm high carbon rich and oxygen rich conventional FEBID Au. It is assumed that the oxygen is bound to the carbon and not to the noble gold. With a 100% cleaning efficiency in the best case all carbon and all oxygen could be removed. In this best case scenario only a 737 nm high deposit of pure gold would remain. In the next step, this issue and the effect of the oxidative enhancement will be investigated.

Adding a small flow of water as an oxidative enhancer during the deposition (chamber pressure: ~7.2 × 10^−5^ mbar), the total height of the deposition decreases. A closer look at the deposited volume contribution reveals that the carbon content of the deposition decreased strongly, and most importantly, the volume of the gold increased significantly. In other words, although the total height of the deposit was only 1700 nm, the gold now contributed 1059 nm to the height of the total volume. In contrast to any post-deposition cleaning procedure, the *in situ* water addition during FEBID of Au can also increase the efficiency of the gold deposition. Only with this small flow of water as an oxidative enhancer, the gold deposition could be increased by 44% (= (1059/737) − 1)*100). The volume contribution of carbon was reduced from 879 nm to 503 nm, which corresponds to a 43% decrease of the carbon volume. In summary, the injection of water as an oxidative enhancer not only results in less carbon in the deposit but also increases the yield of the gold deposition.

It is long known that in condensed and adsorbed molecules the reactivity under electron irradiation may be quenched or enhanced by the surrounding medium^52^. It is reasonable that the chemical reactivity of the gold precursor and the pathway of electron-induced decomposition may change when aggregated with water molecules. The molecular aggregates may exhibit new electronic and vibrational interaction pathways that facilitate the high purity of our deposits^53^.

The astonishing fact that more Au is deposited in the presence of water than with conventional FEBID is explained by the fact that more gold precursor molecules must be decomposed in the presence of water. Through the presence of water, the dissociation cross section for electrons has changed and the dissociation energy of the Au precursor is modified. Therefore, we propose that this higher deposition yield is due to the simple fact that more precursor is adsorbed on the surface. This corresponds to the fact that more gold precursor molecules can stick to the surface of the deposition area—precursors that come from either surface diffusion or from the gas phase. It is speculated that the presence of water facilitates not only the electron-induced oxidation of carbonaceous deposits but also the electron-induced oxidation of the adsorbed organic ligands (trifluoroacetylacetonate and its fragments) and of the residual gas components with high molecular mass. This will free up more surface sites for the adsorption (and later decomposition) of gold precursor molecules. This model is consistent with the observed effects of higher gold deposition yield and higher gold purity of the deposits. Adding more water may further pronounce this effect.

As more water was added (chamber pressure: ~1.2 × 10^−4^ mbar), the deposited gold volume and deposition yield further increase. With gold contributing to 1286 nm of the total height, this is equivalent to a 75% increase in the gold deposition yield. At the same time, the carbon content decreases and contributes to only 144 nm of the height. This is equivalent to an 84% reduction of carbon in the deposited volume. It should be noted that carbon, along with oxygen, decreases. This confirms the previous assumption that oxygen is bound to the carbon. If the oxygen would be bound to gold—which is theoretically possible in gold oxide—then an increase in the gold volume would also lead to an increase in the oxygen volume. This is not the case.

In addition, the used precursor contains oxygen as a ketone group, and this double bond strongly bonds the oxygen to carbon. The electron-induced oxidative removal of carbon (with the oxygen bound to it) results in free adsorption sites on the surface. Because there are still enough gold precursor entering either from the gas phase or by the surface diffusion, this means that the gold deposition yield increases, and, at the same time, the carbon contribution decreases. As shown in Fig. 5, the moderate water injection, leading to a chamber pressure of ~1.2 × 10^−4^ mbar, has led to the highest gold deposition yield observed in this study. This coincides with an 84% reduction of carbon.

However, the oxidative removal of the carbon (and the oxygen bonded to carbon) is possible only if the injected water is also available on the surface. Hence, it is assumed that water must occupy surface sites to chemically react with the carbon- and oxygen-containing surface species. This, unfortunately, also means that water can block surface sites for the adsorption of the gold precursors. Therefore, in the proposed model, an excessive injection of water will lead to a further decrease in carbon contamination but will also reduce the gold deposition yield. This was in fact observed for water injection, leading to a chamber pressure of 1.7 × 10^−4^ mbar and onwards. A similar precursor competition issue was reported for focused ion beam-induced co-deposition from W(CO)_6_ and C_10_H_8_ precursors^54^. Although the above reported work used different molecules than this work, a metalorganic gold precursor and water follows same rationale which is equivalent for both experiments. Both works indicate the validity of the precursor competition mechanism.

For additional water injection (chamber pressure above 1.2 × 10^−4^ mbar), the volume of the gold deposit did not increase any further but rather started to decrease. However, the volume of the deposited carbon still decreased further, which confirms even better cleaning efficiency at a high water pressure. As the water pressure is increased, the excess amount of water on the surface blocks the surface diffusion or incoming adsorption from the gas phase. The gold precursor cannot occupy as many surface sites, and the deposition yield goes down. At this point, it is reasonable to assume that the precursor coverage on the surface is no longer as high as with 1.2 × 10^−4^ mbar. Compared with the water injection up to a chamber pressure of ~1.2 × 10^−4^ mbar, the gold deposition yield was clearly decreased by 10% (1.7 × 10^−4^ mbar) and 27% (2.2 × 10^−4^ mbar), respectively. However, in contrast to the conventional FEBID without an oxidative enhancer, the injection of water to the highest chamber pressure of 2.2 × 10^−4^ mbar still yielded a 28% (944/737) increase in the gold deposition yield. However, excessive water injection further promoted the cleaning efficiency from carbonaceous contaminations of the deposit. Whereas the carbon deposition still contributed to 144 nm of the weight of the deposit, further water injection led to a decrease to 121 nm (1.7 × 10^−4^ mbar) and 50 nm (2.2 × 10^−4^ mbar). Compared with the water injection for the best gold deposition yield (1.2 × 10^−4^ mbar), the further increase in the water injection led to a further decrease in carbon by −16% (1.7 × 10^−4^ mbar) and even −65% (2.2 × 10^−4^ mbar). This confirms the beneficial aspect of high water coverage on the surface. Both effects—the lower gold deposition yield and the increased purity—are consistent with the proposed model.

In summary, we have demonstrated a novel technique that can yield highly pure FEBID Au and that increases the yield of the gold deposition. This novel approach allows the deposition of highly pure Au, *in situ* with the help of water injection, and without any post-deposition purification approach. The high purity of the structure was verified via SEM, TEM EDX and TEM EELS. The conductivity of the FEBID Au obtained is the highest FEBID directly deposited conductivity that has been achieved. In addition, a model has been proposed, based on the deposited volumes and chemical compositions, that explains the mechanism of water as an oxidative enhancer. As predicted by this model, the highest water injection (2.2 × 10^−4^ mbar) yielded the best purification with 94% removal of carbon contamination and a 28% increase in the gold deposition yield relative to the conventional (water-free) Au-FEBID. The best increase in the gold deposition yield was by 75% relative to conventional (water-free) Au-FEBID, which was observed for medium water injection. The present study has provided valuable insight into the mechanisms of *in situ* purification. The purification of gold *in situ* during deposition has laid the foundation for future applications involving low-resistivity electrical interconnects, 3D plasmonic structures and biomolecule immobilization.

## Methods

FEBID experiments were performed inside an oil-free Zeiss Leo 1530VP scanning electron microscope with a custom-made multi-nozzle gas injection system (GIS). Commercially available dimethyl gold (III) trifluoroacetylacetonate precursor was used to fabricate Au samples. The gold precursor and the water were injected via two separate nozzles. Both nozzles are cylindrical in shape, with an inner diameter of 400 μm. The Au nozzle was approximately 300 μm above the surface of the substrate. The deposition location was approximately 1 mm from the centre of the nozzle aperture. The water nozzle was placed 250 μm from the deposition spot horizontally and 100 μm vertically. Both nozzles were inclined ~35° to the surface and mounted in opposite directions, pointing to the same deposition spot. During the deposition, the gold precursor reservoir was heated to ~40 °C. The base pressure of the chamber was ~6.7 × 10^−7^ mbar. For Au deposition, the gold precursor influx resulted in a working pressure of ~1.9 × 10^−5^ mbar. This pressure was also maintained during experiments with water injection. In the case of water injection, the chamber pressure increased from 7.2 × 10^−5^ mbar to 2.2 × 10^−4^ mbar owing to the selected injection flow.

Gold deposition was performed after pumping the system chamber for at least 12 h. Reduced area scan mode with a scan speed 6 was used to perform square deposition with a ~5 nm point-to-point pitch. TEM investigated samples were also fabricated using the same conditions while the precursor was at room temperature. During line deposition, a dwell time of 200 μs and 5 nm point-to-point pitch were used. Also, a Raith Elphy Plus pattern generator was utilized to control the scan procedure of the electron beam. An acceleration voltage of 3 kV and a beam current of 4 nA was used for all oxidative assisted FEBID deposition.

The ultrasonically cleaned n-doped Ge substrate with a native oxide of ~2 nm was used to deposit Au structures for both SEM & TEM analysis. An n-type Si substrate with wet thermal oxide of 90 nm was used for the deposition of Au structures for I-V measurements. There were no signification changes observed in growth rate on Si and Ge substrates.

EDX analysis was performed with a different instrument—a Zeiss Neon 40Esb cross-beam microscope—at a base pressure of ~2 × 10^−6^ mbar. EDX was performed at 5 kV, with an Oxford Instruments EDS 7427 detector. The samples for SEM analysis were briefly exposed (<2 min) to cleanroom air during the transfer from the Zeiss Leo 1530VP SEM to the Zeiss Neon SEM. TEM characterization was performed using an FEI Tecnai F20 microscope equipped with an EDAX detector for energy dispersive spectroscopy and a Fischione 3000 detector for high-angle annular dark field imaging at an acceleration voltage of 200 kV and a GATAN GIF Tridiem EELS detection system. Four-point probe electrical characterization of horizontal NWs was carried out, using an Agilent 4155B semiconductor parameter analyser. AFM measurements were conducted, using a Veeco/Bruker Dimension 3000 atomic force microscope. During the transfer of the TEM samples from the cleanroom to the TEM facility, these samples were exposed to the atmosphere for less than 2 h.

## Additional Information

**How to cite this article**: Shawrav, M. M. *et al*. Highly conductive and pure gold nanostructures grown by electron beam induced deposition. *Sci. Rep.* **6**, 34003; doi: 10.1038/srep34003 (2016).

## Supplementary Material

Supplementary Information

## Figures and Tables

**Table 1:** SEM and TEM comparison of atomic composition.

Treatment\Element	C (at. %)	O (at. %)	Au (at. %)
SEM-EDX	4–8	5–12	81–92
TEM EELS	11	7	82

## References

[b1] Do M. T. . Fabrication and Characterization of Large-Area Unpatterned and Patterned Plasmonic Gold Nanostructures. Journal of Electronic Materials 45, 2347–2353, 10.1007/s11664-015-4291-6 (2016).

[b2] Gong C. & Leite M. S. Noble Metal Alloys for Plasmonics. ACS Photonics 3, 507–513, 10.1021/acsphotonics.5b00586 (2016).

[b3] Han Q. . Ag-Au alloy nanoparticles: Synthesis and *in situ* monitoring SERS of plasmonic catalysis. Sensors and Actuators, B: Chemical 231, 609–614, 10.1016/j.snb.2016.03.068 (2016).

[b4] Yavas O., Ochiai C., Takai M., Hosono A. & Okuda S. Maskless fabrication of field-emitter array by focused ion and electron beam. Applied Physics Letters 76, 3319–3321 (2000).

[b5] Shedd G. M., Lezec H., Dubner A. D. & Melngailis J. Focused ion beam induced deposition of gold. Applied Physics Letters 49, 1584–1586, 10.1063/1.97287 (1986).

[b6] Van Dorp W. F. & Hagen C. W. A critical literature review of focused electron beam induced deposition. Journal of Applied Physics 104, 10.1063/1.2977587 (2008).

[b7] Utke I., Hoffmann P. & Melngailis J. Gas-assisted focused electron beam and ion beam processing and fabrication. Journal of Vacuum Science and Technology B: Microelectronics and Nanometer Structures 26, 1197–1276, 10.1116/1.2955728 (2008).

[b8] De Teresa J. M. & Fernández-Pacheco A. Present and future applications of magnetic nanostructures grown by FEBID. Applied Physics A: Materials Science and Processing 117, 1645–1658, 10.1007/s00339-014-8617-7 (2014).

[b9] De Teresa J. M. & Córdoba R. Arrays of densely packed isolated nanowires by focused beam induced deposition plus Ar+ milling. ACS Nano 8, 3788–3795, 10.1021/nn500525k (2014). 24645869

[b10] Córdoba R., Han D. S. & Koopmans B. Manipulating the switching in modulated iron nanowires grown by focused electron beam induced deposition. Microelectronic Engineering 153, 60–65, 10.1016/j.mee.2016.01.032 (2016).

[b11] Gabureac M. S., Bernau L., Boero G. & Utke I. In *Nanotechnology 2011: Electronics, Devices, Fabrication, MEMS, Fluidics and Computational - 2011 NSTI Nanotechnology Conference and Expo, NSTI-Nanotech 2011.* 226–229.

[b12] Córdoba R. . Nanoscale chemical and structural study of co-based FEBID structures by STEM-EELS and HRTEM. Nanoscale Res. Lett. 6, 1–6, 10.1186/1556-276X-6-592 (2011). PMC3237113 22085532

[b13] Liu Z. Q., Mitsuishi K. & Furuya K. The growth behavior of self-standing tungsten tips fabricated by electron-beam-induced deposition using 200 keV electrons. Journal of Applied Physics 96, 3983–3986, 10.1063/1.1788844 (2004).

[b14] Plank H., Haber T., Gspan C., Kothleitner G. & Hofer F. Chemical tuning of PtC nanostructures fabricated via focused electron beam induced deposition. Nanotechnology 24, 10.1088/0957-4484/24/17/175305 (2013). 23571599

[b15] Donev E. U. & Hastings J. T. Electron-beam-induced deposition of platinum from a liquid precursor. Nano Letters 9, 2715–2718, 10.1021/nl9012216 (2009). 19583284

[b16] Gavagnin M. . Free-standing magnetic nanopillars for 3D nanomagnet logic. ACS Applied Materials and Interfaces 6, 20254–20260, 10.1021/am505785t (2014). 25296008 PMC4251043

[b17] Rührig M., Porthun S. & Lodder J. C. Magnetic force microscopy using electron-beam fabricated tips. Review of Scientific Instruments 65, 3224–3228, 10.1063/1.1144554 (1994).

[b18] Gavagnin M. . Magnetic force microscopy study of shape engineered FEBID iron nanostructures. Physica Status Solidi (A) Applications and Materials Science 211, 368–374, 10.1002/pssa.201330114 (2014).

[b19] Utke I. . Focused electron beam induced deposition of high resolution magnetic scanning probe tips. Making Functional Materials with Nanotubes 706, 307–312 (2002).

[b20] Córdoba R. . Giant anomalous Hall effect in Fe-based microwires grown by focused-electron-beam-induced deposition. Journal of Physics D: Applied Physics 45, 10.1088/0022-3727/45/3/035001 (2012).

[b21] Koops H. W. P., Weiel R., Kern D. P. & Baum T. H. High resolution electron beam induced deposition. Journal of Vacuum Science & Technology B 6, 477–481 (1988).

[b22] Fowlkes J. D., Doktycz M. J. & Rack P. D. An optimized nanoparticle separator enabled by electron beam induced deposition. Nanotechnology 21, 10.1088/0957-4484/21/16/165303 (2010). 20351412

[b23] Burbridge D. J., Crampin S., Viau G. & Gordeev S. N. Strategies for the immobilization of nanoparticles using electron beam induced deposition. Nanotechnology 19, 10.1088/0957-4484/19/44/445302 (2008). 21832725

[b24] Bresin M., Nehru N. & Hastings J. T. Focused electron-beam induced deposition of plasmonic nanostructures from aqueous solutions. Proceedings of SPIE 8613, Advanced Fabrication Technologies for Micro/Nano Optics and Photonics VI, 861305, doi: 10.1117/12.2003055 (2013).

[b25] Dhawan A., Gerhold M., Russell P., Vo-Dinh T. & Leonard D. Fabrication of metallic nanodot structures using focused ion beam (FIB) and electron beam-induced deposition for plasmonic waveguides. Proceedings of SPIE 7224, Quantum Dots, Particles, and Nanoclusters VI, 722414, doi: 10.1117/12.809927 (2009).

[b26] Shawrav M. M. . Mask-free prototyping of metal-oxide-semiconductor devices utilizing focused electron beam induced deposition. Physica Status Solidi (A) Applications and Materials Science 211, 375–381, 10.1002/pssa.201330133 (2014).

[b27] Höflich K., Yang R. B., Berger A., Leuchs G. & Christiansen S. The direct writing of plasmonic gold nanostructures by electron-beam- induced deposition. Advanced Materials 23, 2657–2661, 10.1002/adma.201004114 (2011). 21538585

[b28] Höflich K., Becker M., Leuchs G. & Christiansen S. Plasmonic dimer antennas for surface enhanced Raman scattering. Nanotechnology 23, 10.1088/0957-4484/23/18/185303 (2012). 22498764

[b29] Utke I. . Polarisation stabilisation of vertical cavity surface emitting lasers by minimally invasive focused electron beam triggered chemistry. Nanoscale 3, 2718–2722, 10.1039/c1nr10047e (2011). 21390361

[b30] Utke I. . Focused electron beam induced deposition of gold. Journal of Vacuum Science & Technology B 18, 3168–3171, 10.1116/1.1319690 (2000).

[b31] Mulders J. J. L., Veerhoek J. M., Bosch E. G. T. & Trompenaars P. H. F. Fabrication of pure gold nanostructures by electron beam induced deposition with Au(CO)Cl precursor: Deposition characteristics and primary beam scattering effects. Journal of Physics D: Applied Physics 45, 10.1088/0022-3727/45/47/475301 (2012).

[b32] Miyazoe H. . Improving the metallic content of focused electron beam-induced deposits by a scanning electron microscope integrated hydrogen-argon microplasma generator. Journal of Vacuum Science and Technology B:Nanotechnology and Microelectronics 28, 744–750, 10.1116/1.3449808 (2010).

[b33] Plank H. . Optimization of postgrowth electron-beam curing for focused electron-beam-induced Pt deposits. Journal of Vacuum Science and Technology B 29, 10.1116/1.3622314 (2011).

[b34] Botman A., Mulders J. J. L., Weemaes R. & Mentink S. Purification of platinum and gold structures after electron-beam-induced deposition. Nanotechnology 17, 3779–3785, 10.1088/0957-4484/17/15/028 (2006).

[b35] Riazanova A. V., Rikers Y. G. M., Mulders J. J. L. & Belova L. M. Pattern shape control for heat treatment purification of electron-beam-induced deposition of gold from the Me 2Au(acac) precursor. Langmuir 28, 6185–6191, 10.1021/la203599c (2012). 22413820

[b36] Mehendale S., Mulders J. J. L. & Trompenaars P. H. F. In *Technical Proceedings of the 2013 NSTI Nanotechnology Conference and Expo, NSTI-Nanotech* 474–476 (2013).

[b37] Mulders J. J. L., Belova L. M. & Riazanova A. Electron beam induced deposition at elevated temperatures: Compositional changes and purity improvement. Nanotechnology 22, 10.1088/0957-4484/22/5/055302 (2011). 21178259

[b38] Córdoba R., Sesé J., De Teresa J. M. & Ibarra M. R. High-purity cobalt nanostructures grown by focused-electron-beam-induced deposition at low current. Microelectronic Engineering 87, 1550–1553, 10.1016/j.mee.2009.11.027 (2010).

[b39] Roberts N. A., Fowlkes J. D., Magel G. A. & Rack P. D. Enhanced material purity and resolution via synchronized laser assisted electron beam induced deposition of platinum. Nanoscale 5, 408–415, 10.1039/c2nr33014h (2013). 23184056

[b40] Belić D. . Direct-write deposition and focused-electron-beam-induced purification of gold nanostructures. ACS Applied Materials and Interfaces 7, 2467–2479, 10.1021/am507327y (2015). 25545798

[b41] Shimojo M., Takeguchi M. & Furuya K. Formation of crystalline iron oxide nanostructures by electron beam-induced deposition at room temperature. Nanotechnology 17, 3637–3640, 10.1088/0957-4484/17/15/003 (2006).

[b42] Landheer K. . Low-energy electron-induced decomposition and reactions of adsorbed tetrakis(trifluorophosphine)platinum [Pt(PF3)4]. Journal of Physical Chemistry C 115, 17452–17463, 10.1021/jp204189k (2011).

[b43] Mehendale S., Mulders J. J. L. & Trompenaars P. H. F. Purification of Au EBID structures by electron beam post-irradiation under oxygen flux at room temperature. Microelectronic Engineering 141, 207–210, 10.1016/j.mee.2015.03.034 (2015).

[b44] Wanzenboeck H. D., Roediger P., Hochleitner G., Bertagnolli E. & Buehler W. Novel method for cleaning a vacuum chamber from hydrocarbon contamination. Journal of Vacuum Science & Technology A 28, 1413–1420, 10.1116/1.3484242 (2010).

[b45] Koops H. W. P., Schössler C., Kaya A. & Weber M. Conductive dots, wires, and supertips for field electron emitters produced by electron-beam induced deposition on samples having increased temperature. Journal of Vacuum Science and Technology B: Microelectronics and Nanometer Structures 14, 4105–4109 (1996).

[b46] Mulders H. In *2nd Annual Meeting of COST Action CM 1301.* (ed. Papp P.) 24 (Chemistry for ELectron-Induced NAnofabrication (CELINA), 2015).

[b47] Mulders H. In FEBIP Workshop. (eds Plank H. & Wanzenböck H.) (2016).

[b48] Mulders J. J. L. & Botman A. The use of Au and Pt x-ray N lines for correct EDX analysis of C in gold and platinum depositions of micro and nano structures. Microsc. Microanal. 15, 1126, 10.1017/S1431927609095385 (2009).

[b49] Koops H. W. P. . Characterization and application of materials grown by electron-beam-induced deposition. Jpn. J. Appl. Phys. 33, 709–717, 10.1143/JJAP.33.7099 (1994).

[b50] Plank H. . Electron-beam-assisted oxygen purification at low temperatures for electron-beam-induced Pt deposits: Towards pure and high-fidelity nanostructures. ACS Applied Materials and Interfaces 6, 1018–1024, 10.1021/am4045458 (2014). 24377304

[b51] Fernndez-Pacheco A., De Teresa J. M., Córdoba R. & Ibarra M. R. Magnetotransport properties of high-quality cobalt nanowires grown by focused-electron-beam-induced deposition. Journal of Physics D: Applied Physics 42, 10.1088/0022-3727/42/5/055005 (2009).

[b52] Ingólfsson O., Weik F. & Illenberger E. The reactivity of slow electrons with molecules at different degrees of aggregation: Gas phase, clusters and condensed phase. International Journal of Mass Spectrometry and Ion Processes 155, 1–68 (1996).

[b53] Michael G., Mark A. R. & Abraham N. Molecular transport junctions: vibrational effects. Journal of Physics: Condensed Matter 19, 103201 (2007).

[b54] Serrano-Esparza I., Córdoba R., Mulders J. J. L., Ibarra M. R. & De Teresaa J. M. Precursor competition in focused-ion-beam-induced co-deposition from W(CO)6 and C10H8. Science Letters Journals 4 (2015).

