# Supplementary material for: Highly conductive and pure gold nanostructures grown by electron beam induced deposition
Source: Sci Rep. 2016 Sep 26;6:34003. doi: 10.1038/srep34003 (PMC5035929; doi:10.1038/srep34003)
Supplement: Supplementary Information [file srep34003-s1.pdf]

Supplemental Information

Highly conductive and pure gold nanostructures grown by electron beam induced deposition

Mostafa M. Shawrav[1+], Philipp Taus[1+], Heinz D. Wanzenboeck[1*], M. Schinnerl[1], M. Stöger-Pollach[2], S.Schwarz[2], A. Steiger-Thirsfeld[2] and Emmerich Bertagnolli[1]

[1]Institute of Solid State Electronics, Vienna University of Technology, Vienna 1040, Austria

[2]University Service Center for Transmission Electron Microscope (USTEM), Vienna University of Technology, Vienna 1040, Austria

[*]Heinz.wanzenboeck@tuwien.ac.at

[+]these authors contributed equally to this work

Supplement 1: a) SEM EDX Map b) a Line scan on a water boosted FEBID Au square structure c) Partial Gold and water precursor pressure corresponding to total pressure

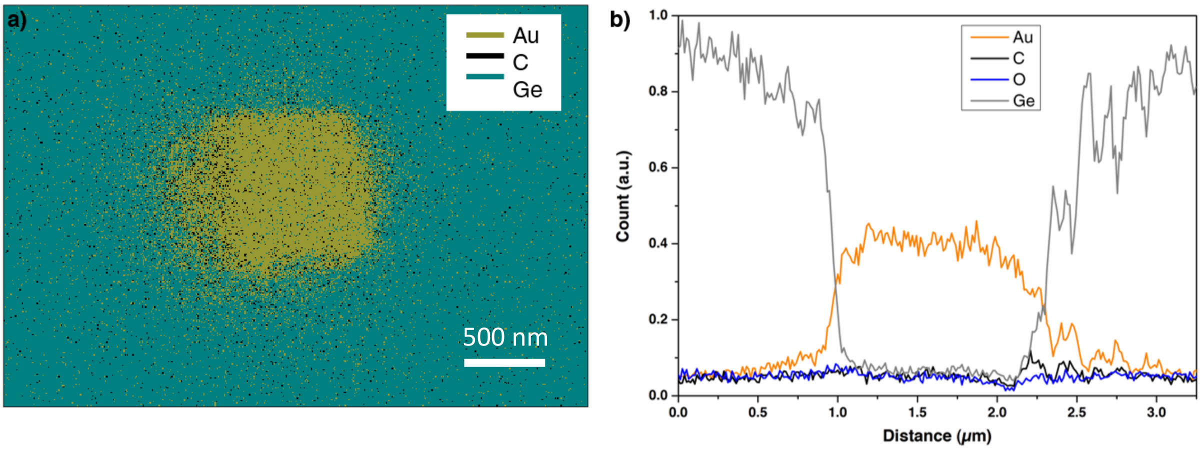

C) Partial gold and water precursor pressure corresponding to the total pressure and ratio. Please note that, we only can access the total chamber pressure. Water and precursor partial pressures are mathematically calculated subtracting gold precursor pressure from total chamber pressure assuming that gold precursor flux was constant. After injecting the gold precursor, a 30 min waiting period was applied to make sure the gold precursor pressure remained constant. After the waiting period, water was injected into chamber.

Total Pressure [mbar]	Au Partial pressure [mbar]	H_2O partial Pressure [mbar]	Nominal ratio of Au-precursor to water $Me_2Au(tfac):H_2O$)
1.90×10^{-5}	1.90×10^{-5}	0	1:0
7.20×10^{-5}	1.90×10^{-5}	5.30×10^{-5}	1:2.8
1.20×10^{-4}	1.90×10^{-5}	1.01×10^{-4}	1:5.3
1.70×10^{-4}	1.90×10^{-5}	1.51×10^{-4}	1:7.9
2.20×10^{-4}	1.90×10^{-5}	2.201×10^{-4}	1:10.5

Supplement 2: a) & b) Monte Carlo Simulation of the electron distribution along z-axis. It shows a complete protection of the Au deposition using a 150 nm Cr layer for electron beam irradiation during the sample handling. c) Bright field TEM Image corresponds to different layer of the deposits. d) Diffraction pattern shows a polycrystalline structure

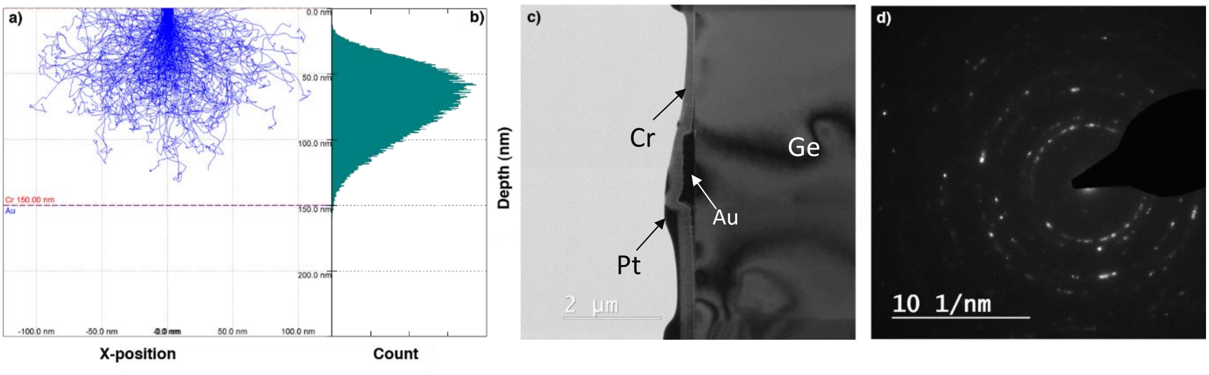

Supplement 3: a) Characteristic x-ray energies identified.

Chemical Element	Characteristic Line	Energy (keV)
**C**	K	0.28
**O**	K	0.53
**Cu**	$L_{\alpha1}$	0.93
**Au**	$M_{\alpha1}$	2.14
**Cr**	$K_{\alpha1}$	5.41
**Cr**	$K_{\beta1}$	5.94
**Fe**	$K_{\alpha1}$	6.40
**Co**	$K_{\alpha1}$	6.93
**Cu**	$K_{\alpha1}$	8.05
**Au**	L_{l}	8.49
**Cu**	$K_{\beta1}$	8.90
**Au**	$L_{\alpha1}$	9.70
**Au**	$L_{\beta3}$	11.43
**Au**	$L_{\beta1}$	11.61
**Au**	$L_{\gamma1}$	13.38

Supplement 3 b) TEM EELS spectrum of the water co-deposited gold square

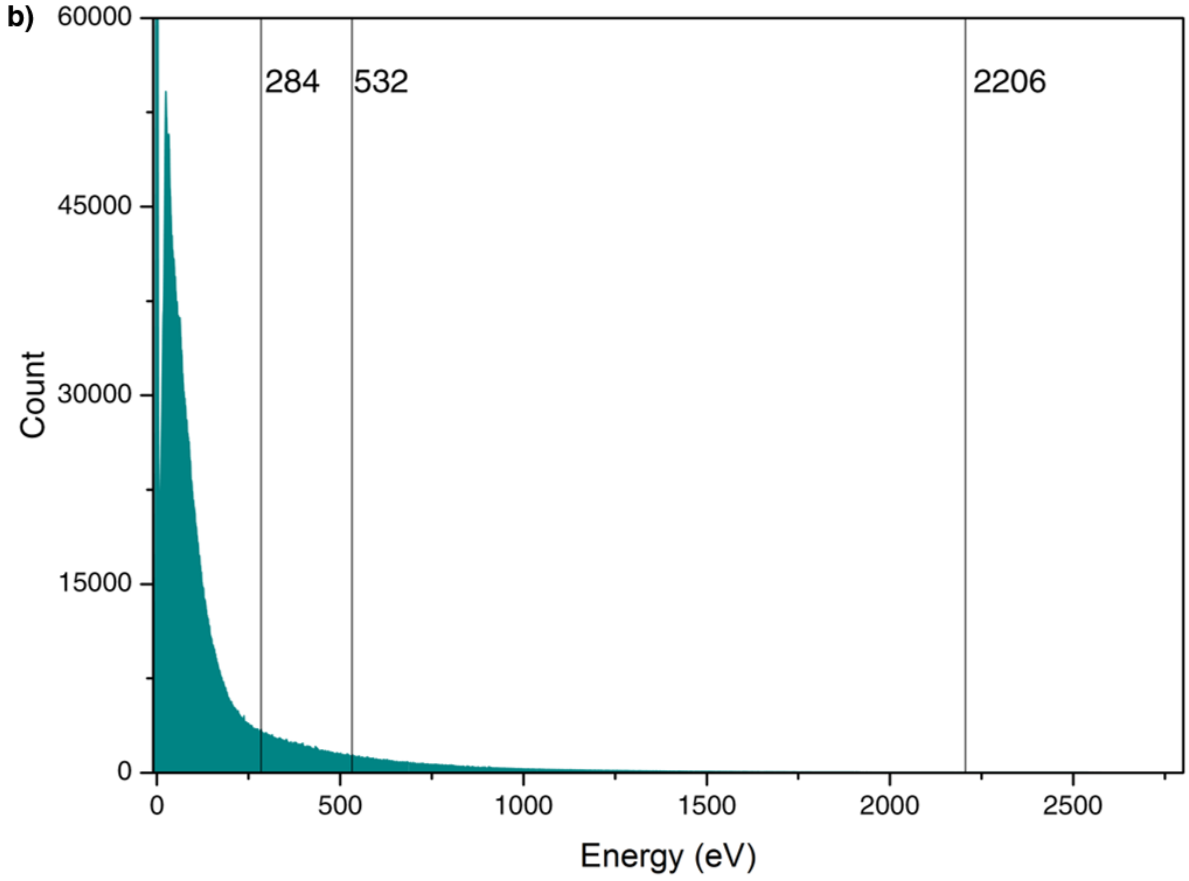

Supplement 4: I-V characteristics of conventional FEBID Au.

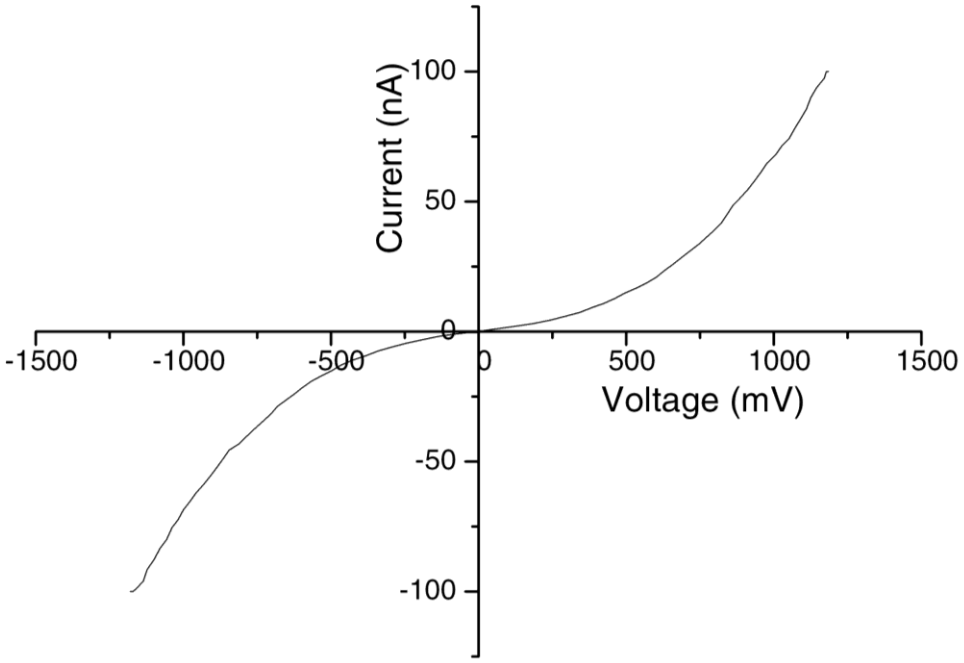

Supplement 5: Specific volume contribution of gold, oxygen and carbon compared to height and atomic % for different pressures. Please note that the left column for each pressure corresponds to the atomic % (not in nanometer).

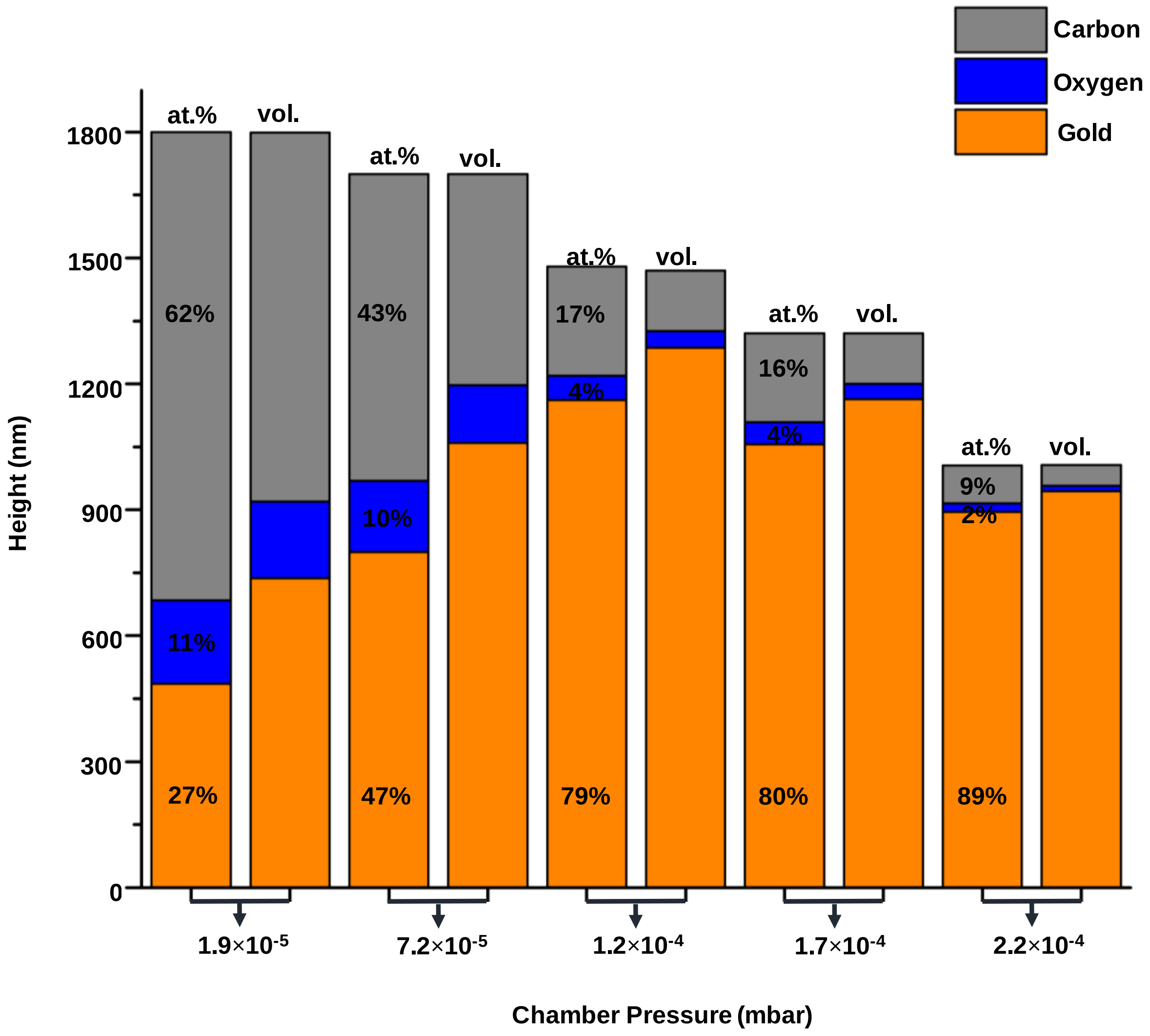

Supplement 6: Calculation for Figure 5 and Supplement 5.

Chamber Pressure (mbar)	Au (at. %)	O (at. %)	C (at. %)
1.9×10^{-5}	27	11	62
7.2×10^{-5}	47	10	43
1.2×10^{-4}	79	4	17
1.7×10^{-4}	80	4	16
2.2×10^{-4}	89	2	9

Table 6-1: Atomic composition (atomic %) obtained from SEM EDX

For volume calculation following equation is used

$$\text{Atomic \% in mol} \times \frac{\text{Molar mass in g/mol}}{\text{Density of material in g/cm3}}$$

$$= \text{Volume in cm3}\ldots..\text{(i)}$$

From Literature

Molar mass of gold :197 g/mol

Molar mass of Carbon: 12

Density of gold: 19.3 g/cm3

Density of Graphite: 2.26 g/Cm3

Molar mass/Density of Oxygen: 6.23 mathematically calculated from oxygen in solid

Example of calculation for Chamber Pressure, 1.9×10^{-5} mbar

Equation (i) was applied for each material. Atomic % obtained from EDX presented in Table 6-1:

For Au: 27 ×(197/19.3): 275.60

For O:11 × 6.23: 68.53

For C: 62 × (12/2.26): 328.60

Therefore, the corresponding vol. % result in:

For Au: 275.60/(275.60+68.54+328.60) × 100 :41%

For O: 68.53/(275.60+68.54+328.60) × 100: 10%

For C: 328.60/(275.60+68.54+328.60) × 100: 49%

After that, the vol. % of the specific height of this pressure (1800nm) was calculated for each material which results in 737 nm for Au, 183 nm for O and 870 nm for C.

The calculation was repeated for each chamber pressure and the result is presented in following table:

Chamber Pressure (mbar)	Nominal ratio of Au-precursor to water	Atomic % from EDX			Volume contribution in %			Volume contribution corresponding to the deposit's total height in nm			Measured Height of the deposit (nm)
	$Me_2Au(tfac):H_2O$	Au	O	C	Au	O	C	Au	O	C	
1.9×10^{-5}	1:0	27	11	62	41	10	49	737	183	879	1800
7.2×10^{-5}	1:3.8	47	10	43	62	8	30	1059	138	503	1700
1.2×10^{-4}	1:6.3	79	4	17	88	3	10	1286	40	144	1470
1.7×10^{-4}	1:8,9	80	4	16	88	3	9	1164	36	121	1320
2.2×10^{-4}	1:11.6	89	2	9	94	1	5	944	13	50	1006

Table 6-2:

Atomic % is obtained from EDX

Volume Contribution in %: Calculated from the above formula in supplement 6

Volume contribution in nm of total height: Specific height of Au, O and C in nm contributing to the total height of the structure

Height : Total height of the structure.
